# Phytoplankton Responses to Bacterially Regenerated Iron in a Southern Ocean Eddy

**DOI:** 10.3390/microorganisms10081655

**Published:** 2022-08-16

**Authors:** Marion Fourquez, Robert F. Strzepek, Michael J. Ellwood, Christel Hassler, Damien Cabanes, Sam Eggins, Imojen Pearce, Stacy Deppeler, Thomas W. Trull, Philip W. Boyd, Matthieu Bressac

**Affiliations:** 1 Institute for Marine and Antarctic Studies, University of Tasmania, Hobart 7004, Australia; 2 Antarctic Climate and Ecosystems CRC, University of Tasmania, Hobart 7004, Australia; 3 Aix Marseille Université, Université de Toulon, CNRS, IRD, MIO UMR 110, 13288 Marseille, France; 4 Australian Antarctic Program Partnership (AAPP), Institute for Marine and Antarctic Studies, University of Tasmania, Hobart 7004, Australia; 5 Research School of Earth Sciences, Australian National University, Canberra 2601, Australia; 6 Marine and Lake Biogeochemistry, Department F.-A. Forel, University of Geneva, 1205 Geneva, Switzerland; 7 Institute of Earth Sciences, University of Lausanne, 1015 Lausanne, Switzerland; 8 Australian Antarctic Division (AAD), Kingston 7050, Australia; 9 National Institute of Water and Atmospheric Research, Wellington 6021, New Zealand; 10 Climate Science Centre, Oceans and Atmosphere, Commonwealth Scientific and Industrial Research Organisation, Hobart 7004, Australia; 11 Laboratoire d’Océanographie de Villefranche, Sorbonne Université, CNRS, 06230 Villefranche-sur-Mer, France

**Keywords:** iron regeneration, particles, Southern Ocean, eddies, vertical supply, Subantarctic

## Abstract

In the Subantarctic sector of the Southern Ocean, vertical entrainment of iron (Fe) triggers the seasonal productivity cycle but diminishing physical supply during the spring to summer transition forces microbial assemblages to rapidly acclimate. Here, we tested how phytoplankton and bacteria within an isolated eddy respond to different dissolved Fe (DFe)/ligand inputs. We used three treatments: one that mimicked the entrainment of new DFe (Fe-NEW), another in which DFe was supplied from bacterial regeneration of particles (Fe-REG), and a control with no addition of DFe (Fe-NO). After 6 days, 3.5 (Fe-NO, Fe-NEW) to 5-fold (Fe-REG) increases in Chlorophyll *a* were observed. These responses of the phytoplankton community were best explained by the differences between the treatments in the amount of DFe recycled during the incubation (Fe-REG, 15% recycled c.f. 40% Fe-NEW, 60% Fe-NO). This additional recycling was more likely mediated by bacteria. By day 6, bacterial production was comparable between Fe-NO and Fe-NEW but was approximately two-fold higher in Fe-REG. A preferential response of phytoplankton (haptophyte-dominated) relative to high nucleic acid (HNA) bacteria was also found in the Fe-REG treatment while the relative proportion of diatoms increased faster in the Fe-NEW and Fe-NO treatments. Comparisons between light and dark incubations further confirmed the competition between picophytoplankton and HNA for DFe. Overall, our results demonstrate great versatility by microorganisms to use different Fe sources that results in highly efficient Fe recycling within surface waters. This study also encourages future research to further investigate the interactions between functional groups of microbes (e.g. HNA and cyanobacteria) to better constraint modeling in Fe and carbon biogeochemical cycles.

## 1. Introduction

Low concentrations of iron (Fe) exert a strong influence on primary productivity across much of the Southern Ocean (SO) [1]. Nevertheless, widespread phytoplankton blooms occur each year due to the resupply of dissolved Fe (DFe) over wide areas of the SO [2]. In early spring, this Fe fertilization is dominated by a one-off pulse of new DFe from the subsurface reservoir through deep winter mixing and entrainment [3,4]. This new DFe is rapidly consumed by the upper ocean biota and, as the mixed layer (ML) depth decreases over the season, the diapycnal diffusion of regenerated DFe (from subsurface biological recycling) becomes a major mechanism to extend the duration of summertime production [3,5,6].

Several studies have investigated how the phytoplankton community responds to transient ML deepening (e.g. [7,8]) but confounding effects have hindered our understanding of the biological responses to different Fe sources. For example, during late summer—when Fe limitation is greatest [9,10,11]—the response of phytoplankton to transient ML deepening is partly controlled by the degree of Fe limitation relative to the light availability [12,13]. Furthermore, changes in vertical mixing can alter predator–prey interactions [14] and the effect of ML deepening on the phytoplankton community then becomes more complex. The marine biota have therefore devised strategies in response to seasonal changes in Fe availability. At the cellular level, the upregulation of Fe transport systems (i.e., [15,16,17]) and substitution with isofunctional Fe-free proteins [18,19,20] increase Fe uptake rates and decrease the metabolic requirements for Fe, respectively. At the community level, intense grazing- and viral-mediated Fe recycling can account for most of the microbial Fe demand and succession in communities will eventually occur [21,22,23,24].

Heterotrophic prokaryotes (hereafter ‘bacteria’) also play a key role in DFe recycling. Particulate Fe (pFe) lost during cell lysis can be solubilized in the upper water column by bacteria, which ultimately replenishes the DFe pool [25] and references herein). This remineralization of pFe by bacteria also occurs at depth, often on sinking or suspended biogenic particles, which resupplies surface waters through vertical mixing [3,6,26]. Therefore, this source relies heavily upon the efficiency of Fe recycling within the microbial loop (termed the ‘ferrous wheel’, [27]) and can drive 50 to >90% of Fe-fueled productivity [22].

Within the ferrous wheel, bacteria are also pivotal in setting Fe bioavailability for the entire microbial community. Indeed, most remineralization of organic material in the ocean is driven by these microorganisms, a process that returns pFe into dissolved forms [26,28] together with Fe-binding ligands [29,30]). Bacteria also represent a large fraction of the biogenic Fe pool and contribute significantly to DFe utilization in the ML [22,28,31]. Rates of DFe regeneration by bacteria [24,28] can effectively meet the Fe requirements of phytoplankton [32]. However, bacterially regenerated sources of DFe may not be bioavailable to all organisms [6]. This raises the following questions: can surface microbial communities access Fe from bacterial activities that occur at depth? If some taxa target the supply of new Fe [6], do others focus on recycled forms? These aspects are of particular importance in oceanic features where external Fe sources are very limited or non-existent, such as in persistent strong eddies in the Subantarctic Zone (SAZ; [33]).

**Figure 1:**
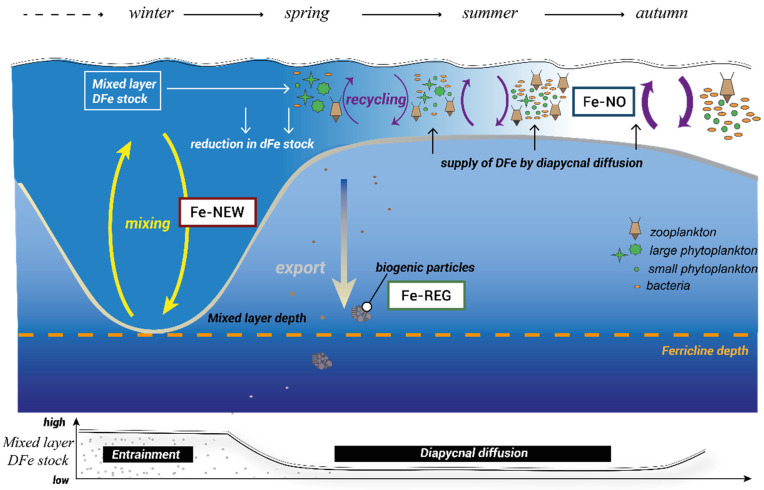
A schematic representation of the seasonal variability in Southern Ocean Fe cycling adapted from [3]. The dominant physical processes over the season are conceptualized at the bottom of the figure with the evolution of DFe inventories in the mixed layer. DFe sources (Fe-NEW, Fe-REG, and Fe-NO) used in this study aimed to represent the seasonal transition of modes of DFe supply from mainly new DFe early in the season (entrainment) to regenerated DFe from the recycling of sinking materials later during the summer (diapycnal diffusion) and to no DFe supply in the autumn.

Eddies are highly variable physical–chemical features in space and time and can become structurally closed. The mechanisms and physical–biogeochemical interactions within eddies are known to modulate phytoplankton productivity and community structure [34]. Physical mechanisms are better constrained but what is less clear is how nutrients cycle within these isolated features to sustain life. Therefore, an eddy is an ideal ‘natural mesocosm’ in which to study the biological response to different Fe inputs. In this study, we tested the response of in-eddy resident microbial communities to differing Fe supply (and Fe-binding ligand) scenarios. From early spring to late summer, the *f*e ratio (i.e., the proportion of Fe uptake from new sources relative to new + regenerated sources; [5]) is expected to decline in concert with the growing dependency of the biota on regenerated DFe ([3], Figure 1). To mimic this supply of subsurface DFe along with the alteration of predator–prey interactions, we simulated changes in the top-down control of phytoplankton stocks through dilution. This approach could lead to a decoupling of the predator–prey link in the ferrous wheel. However, it was a necessary step toward investigating the physiological changes, community shifts, and competitive interactions among the different functional groups (phytoplankton and bacteria) to different DFe sources. Hence, we followed and compared the biological responses of the surface community to the following “treatments” (Figure 1): the supply of subsurface upwelled new DFe (Fe-NEW), diffusive supply from subsurface waters with regenerated DFe (Fe-REG), and ambient surface DFe with minimal new input and high DFe recycling (Fe-NO).

## 2. Material and Methods

### 2.1. Oceanographic Settings

The study was carried out in April 2016 aboard the RV *Investigator* in the Subantarctic Zone of the Southern Ocean (EDDY cruise, part of the V02-IN2016 voyage from 11 March to 17 April 2016) at the center of a cyclonic/cold-core eddy (50.4° S, 147.1° E; 190 km in diameter; Supplementary Figure S1). In late summer 2016, an isolated eddy detached from the Subantarctic Front [35] and was characterized by an extremely low DFe inventory [36] and low primary productivity [37]. This eddy was sampled in the middle of its lifetime during late summer/earlier fall when biological production is expected to be particularly sensitive to the vertical entrainment of new DFe [10] and when microbial residents are acclimated to very low Fe concentrations.

### 2.2. Experimental Set-Up

We used a two-step approach to test how inputs of Fe ligands from the remineralization of subsurface particles influence surface microbial communities: (1) collection and preparation of DFe sources and (2) dilution of surface seawater with the DFe sources. The experimental manipulation behind each step is detailed in the Supplementary Materials (Supplementary Figure S2). We chose this approach to represent the hypothetical transition of modes of Fe supply (Figure 1) from mainly new DFe early in the season (entrainment), regenerated DFe from the remineralization by bacteria of subsurface particles in summer (diapycnal diffusion), and no supply of DFe (dominance of DFe recycling in surface, Figure 1). The shipboard bottle incubation experiment was performed with a natural microbial assemblage sampled within the eddy. Additional experiments were conducted on seawater profiles (15–300 m) collected at the “center” (50.4° S, 147.1° E) and at the “edge” of the eddy (49.7° S, 146.4° E) to compare the bacterial activities inside and outside the eddy. All manipulations were conducted under strict trace metal cleaning conditions in a clean container and under a Class 100 laminar flow hood to avoid unwanted contamination. Trace metal cleaning procedures for labware (including the incubation bottles) followed the Geotraces Cookbook [38]. Moreover, to minimize risks of the potential contamination of samples with metals or dissolved organic matter as an artifact of filtration in preparation for source of DFe, seawater was filtered at a very low pressure (<5 Hg).

### 2.3. Collection and Preparation of DFe Sources

We collected particles at 150 m depth at the center of the eddy (Supplementary Figure S1) by in situ filtration (McLane Research Laboratories in situ pumps). A total of 345 L of seawater was passed through acid-leached 1.0-µm polycarbonate (PC) filters (142 mm diameter). The subsurface particles were gently resuspended in a 10 L High Density Polyethylene (HDPE) acid-washed bottle containing 7 L of <0.2-µm seawater (acid-cleaned Supor Acropak 200 capsule filter) collected at the same depth, resulting in an approximately 50-fold concentration factor of particles. For 6 days, the particles with their attached bacteria were incubated in the dark (to avoid the photochemical breakdown of ligands) under gentle agitation and at the in situ temperature of 7 °C. We assumed that (as we concentrated the particulate fraction) mainly attached bacteria were involved in the degradation of the particulates and the release of DFe and ligands into the dissolved phase. The efficiency of bacterial remineralization was assessed over time by measuring the total and free-living bacterial production (BP) along with changes in nutrient (ammonium (NH_4_), nitrite (NO_2_), nitrate (NO_3_), phosphate (PO_4_), and silicate (Si)) concentrations, including DFe. Following 6 days of incubation, seawater containing regenerated Fe was filtered onto a 0.2-µm acid-cleaned PC filter to remove bacteria for the shipboard incubations experiment (see below) and to mimic the supply of regenerated DFe from subsurface materials.

Six days following our first visit, the sampling site was visited a second time to collect seawater below the mixed-layer depth (100 m) and at the surface. To mimic the supply of new DFe by entrainment, seawater was sampled at a 150 m depth from trace metal cleaned Niskin bottles attached to an autonomous rosette. Upon retrieval, the Niskin bottles were transferred into a clean container and seawater was directly filtered from the Niskin bottles through an acid-cleaned 0.2-µm capsule filter (Supor Acropak 200, Pall; Supplementary Figure S2).

Hence, “new Fe” is the result of several unidentified processes that occur at depth (e.g., grazing, virus attack, cell lysis and release of organic compounds, remineralization etc.) while “regenerated DFe” originates from one particular process that we have isolated: the remineralization of pFe by particle-attached bacteria and the resulting release of DFe.

For the surface DFe source, seawater was collected at a 5 m depth using a towed fish system, drawn onboard using an air-driven Teflon diaphragm pump and directly filtered through an acid-cleaned 0.2-µm capsule filter (Supor Acropak 200, Pall).

### 2.4. Incubation of Surface Microbial Communities

Microbial communities of phytoplankton and bacteria were collected at the same time and using the same procedure as for the collection of the ambient DFe source (excluding filtration). The incubation experiment was conducted in acid-washed 1 L round polycarbonate bottles in which 375 mL of surface seawater was mixed with (i) <0.2-µm 375 mL of the new DFe source (Fe-NEW treatment), (ii) <0.2-µm 375 mL of the regenerated DFe source (Fe-REG treatment), and (iii) <0.2-µm 375 mL of the surface DFe source (Fe-NO treatment). This resulted in a systematic dilution of the surface community by 50% in all treatments (Supplementary Figure S2). Twelve independent replicates per treatment were covered with shade cloth (73 ± 5% of surface irradiance) and placed in an on-deck incubator with continuous seawater supply (9.9 ± 1.1 °C) for up to 6 days. Three independent replicates were harvested at days 0, 2, 4 and 6 for biological and chemical parameters.

Additional experiments we conducted in the dark to assess whether dissolved organic carbon (DOC), DFe or both were in limiting concentrations for heterotrophic activities by prokaryotes. To do so, 250 mL of Fe-NO seawater (125 mL unfiltered surface seawater +125 mL of <0.2 surface water) was dispensed into 300 mL PC bottles and amended either with 1 nM FeCl_3_ (“+Fe”), 60 µM of organic carbon (as 10 µM of trace metal cleaned glucose, “+C”) or a combination of both (“+Fe+C”). Note that in the “+Fe+C” condition, 16.6 µmol Fe was added per mol C^−^^1^ to match the bacterial Fe quota observed for Fe-replete bacterial cultures [39]. Control incubations with Fe-NO and Fe-REG waters (no amendment) were also conducted under similar conditions. Supplementary Figure S2 summarizes the experimental set-up. Subsamples from each triplicate for bacterial abundance and production were taken at days 0, 2, 4 and 6 for comparison with light incubations.

### 2.5. Biological Metrics

#### 2.5.1. Cell Abundances

Enumeration of pico- and nanophytoplankton, cyanobacteria and bacterial cells was done by flow cytometry with similar methods and instrumentation as described in [40]. Briefly, 4.5 mL subsamples were fixed with glutaraldehyde (0.5% final concentration) in the dark at 4 °C for 20 min, flash-frozen in liquid nitrogen, and stored at −80 °C until analysis. High (HNA) and low nucleic acid content (LNA) prokaryote cells were differentiated by their respective signatures in green fluorescence versus side scatter bivariate plots. Autotrophic cell populations were separated into regions based on their autofluorescence in red (FL3) versus orange (FL2) bivariate scatter plots. Cyanobacteria were determined from their high FL2 and low FL3 fluorescence. Pico- and nanophytoplankton communities were determined from their relative cell size using side scatter versus FL3 bivariate scatter plots.

#### 2.5.2. Pigments Composition

For the analysis of the photosynthetic pigments, samples (400–600 mL) were filtered onto 25-mm glass fiber filters (GF/F, Whatman, Maidstone, UK), which were then flash frozen in liquid nitrogen and stored at −80 °C. Concentrations of chlorophyll *a* (Chl *a*) and other pigments were determined by High Performance Liquid Chromatography (HPLC) following the procedure detailed in [41]. Pigments were regrouped into indices using diagnostic pigments (DP = alloxanthin (Allo) + 19′-hexanoyloxyfucoxanthin (Hex) + 19′-butanoyloxyfucoxanthin (But) + fucoxanthin (Fuco) + zeaxanthin (Zea) + chlorophyll b (Chlb) + peridinin (Peri)). Several of these pigments are reliable indicators of specific phytoplankton groups and taxa [42,43,44,45]. Hence, they were used to follow the temporal evolution of different size-fractions by looking at their proportion relative to total DP (Supplementary Table S1): the picophytoplankton fraction (PPF = (Zea + Chlb)/DP), the nanophytoplankton fraction (NPF = (Hex + But + Allo)/DP), and the microphytoplankton fraction (MPF = (Fuco + Peri)/DP). Among those size-fractions, specific taxa were also identified such as diatoms (Fuco) and haptophytes (Hex) (Kramer and Siegel 2019). Note that the picophytoplankton fraction (PPF = (Zea + Chlb)/DP) and the nanophytoplankton fraction (NPF = (Hex + But + Allo) compared well with pico- (R^2^ = 0.97, *n* = 9) and nanophytoplankton (R^2^ = 0.84, *n* = 9) cell abundances measured by flow cytometry.

#### 2.5.3. Photochemical Efficiency

The maximum quantum yield of photosystem II (F_v_/F_m_) was determined on the basis of variable Chl *a* fluorescence using a Fast Repetition Rate fluorometer (Chelsea Technologies Group Fast Ocean Sensor). Triplicate samples (20 mL) were taken from each incubation bottle and dark-acclimated for 30 min prior the measurements to allow for the full oxidation of all photosystem II reaction centers. F_v_/F_m_ (where F_v_ = F_m_ − F_0_) was derived from F_0_ and F_m_, which respectively refer to the minimum and maximum fluorescence in the dark-acclimated state.

#### 2.5.4. Bacterial Production

BP was estimated by the ^3^H-Leucine incorporation method [46] adapted to microcentrifuge tubes by [47]. Briefly, 1.5 mL samples were incubated in the dark at in situ temperatures for 2–3 h with a mixture of radioactive (L-[3,4,5-3H(N)] PerkinElmer, specific activity 123.8 mCi.mol^−1^) and nonradioactive leucine (20 nM final concentration). Samples were run with two technical replicates and one trichloroacetic acid (TCA; Sigma)-killed control (5% [*v/v*] final concentration). To terminate leucine incorporation, 200 µL of 50% TCA was added to all but the control tubes. Then, samples were centrifuged at 16,000× *g* for 10 min and the supernatant was discarded. The resultant precipitated cells were washed with 1.5 mL of 5% TCA and vortex mixed. Samples were centrifuged one more time (16,000× *g* for 10 min) and the supernatant was removed. Subsequently, 1.5 mL of UltimaGoldTM uLLt (PerkinElmer) was added to each tube, mixed, and allowed to sit for >24 h before the radioactivity was counted onboard with a Hidex 300 SL Liquid Scintillation Counter. The linearity of leucine incorporation was tested in parallel. Details for the calculation can be found in [40]).

### 2.6. Chemical Analyses

Dissolved inorganic macronutrients were analyzed directly on board with a segmented flow analyzer (AAIII HR Seal Analytical) according to [48]. Detection limits were 0.02 μM for P, 0.02 μM for N, and 0.2 μM for Si. The analyses for Fe speciation were done upon our return. DFe was analyzed by flow injection with online preconcentration and chemiluminescence detection (adapted from [49]). The detection limit was 40 pM and the accuracy of the method was controlled by analyzing the SAFe S (0.11 ± 0.04 nmol kg^−1^ (*n* = 3); consensus value 0.093 ± 0.008 nmol kg^−1^), and SAFe D1 (0.66 ± 0.06 nmol kg^−1^ (*n* = 4); consensus value 0.67 ± 0.04 nmol kg^−1^) standards. Organic speciation for Fe was measured by Competitive Ligand Exchange–Adsorptive Cathodic Stripping Voltammetry as described in [50].

### 2.7. Statistical Analyses

All data are given as the means and standard deviations of three biological replicates or as otherwise indicated. All statistical comparisons were performed using a one-way analysis of variance (ANOVA) or the Student’s *t*-test. Differences were considered statistically significant at *p* < 0.05. When significant differences were encountered, a posteriori Holm–Sidak tests were performed (level of significance set at 0.05). All statistical analyses were performed using SigmaPlot 11.0 (SysStat Software, San Jose, CA, USA) or R software [51].

## 3. Results

### 3.1. Bacterial Remineralization of Fe from Subsurface Particles

**Figure 2:**
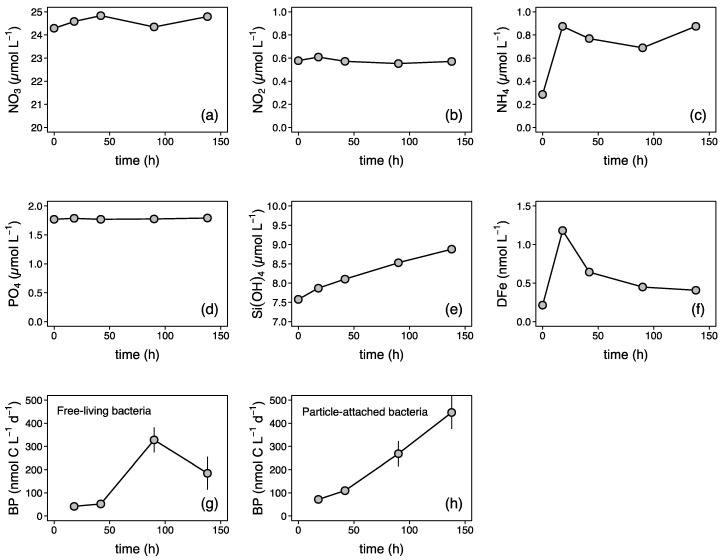
Time evolution of dissolved (**a**) nitrate (NO_3_), (**b**) nitrite (NO_2_), (**c**) ammonium (NH_4_), (**d**) phosphate (PO_4_), (**e**) silicate (Si(OH)_4_), and (**f**) dissolved iron (DFe) concentrations, and bacterial production (BP) by (**g**) free-living and (**h**) particle-attached heterotrophic bacteria during the regeneration of subsurface particles (Section 2.2). Particle-attached BP values were obtained by subtracting the free-living (<1-µm) from the total (unfiltered) BP rates.

We used freshly regenerated DFe from subsurface particles to mimic resupply via diapycnal diffusion (Fe-REG treatment, see Figure 1). Using the particulate Fe (pFe) concentration measured at 150 m depth (0.025 nM, data not shown) in conjunction with the particle concentration factor of 50, we estimated that 16% of the pFe was transferred to the dissolved phase after 6 days. In the natural environment, the partitioning between particulate and dissolved Fe phases can result from both biotic and abiotic dissolution processes [6]. Here, there are several lines of evidence to suggest that biotic actions were at play. A continuous increase in BP rates for particle-attached bacteria over the course of the regeneration incubation confirms that they were metabolically active (Figure 2). An increase in the NH_4_^+^ concentration (Figure 2c), the most commonly regeneration product by bacteria [52], further confirms that remineralization took place rapidly after the resuspension of the particles. There was also indirect evidence of both rapid bacterial release and then consumption balanced with release of DFe (Figure 2f).

### 3.2. Biological Responses to Fe Sources

In this study, we investigated the responses of both phytoplankton and bacteria to different DFe sources. The biological responses were ultimately driven by a range of mechanisms which can be broadly split between phototrophic (influenced by Fe) and heterotrophic (influenced by both Fe and C) responses.

#### 3.2.1. Responses of Phototrophs

**Figure 3:**
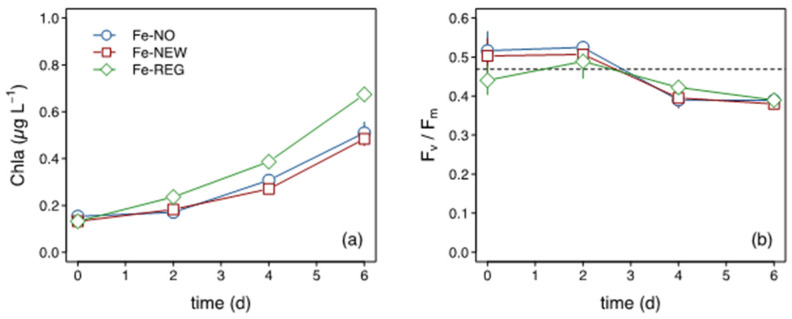
Time course of (**a**) Chl *a* concentration and (**b**) F_v_/F_m_. The horizontal dotted line in (**b**) corresponds to in situ F_v_/F_m_ at the start of the incubation.

Chlorophyll *a* (Chl *a*) concentrations increased within 2 days, and by the end of the incubation a 3.5- (Fe-NO, Fe-NEW) to 5-fold (Fe-REG) increase in Chl *a* was measured (Figure 3a). The Fe-REG treatment showed the greatest increase in Chl *a*, which was significantly different (Student’s test, *p* < 0.01) to the two other treatments. In contrast, no differences in Chl *a* were found between the Fe-NO and Fe-NEW treatments. A decrease in F_v_/F_m_ (Figure 3b) was also observed from day 2 but there was no significant difference between treatments.

**Figure 4:**
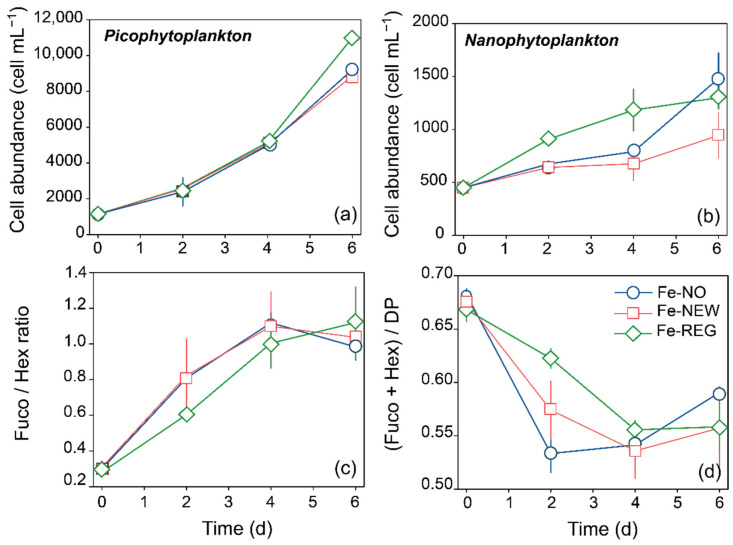
Time course of (**a**) picophytoplankton and (**b**) nanophytoplankton cell abundance measured by flow cytometry, of (**c**) the ratio of diatoms to haptophytes (Fuco/Hex ratio), and the (**d**) relative proportion of pigments assigned to diatoms and haptophytes (Fuco + Hex/DP). Error bars represent the standard deviation of triplicate incubation bottles.

Although the initial phytoplankton community was dominated by nanophytoplankton (i.e., 2–20 µm, 64 ± 1%), the increase in Chl *a* by day 6 was mainly due to an increase in the abundance of the picoplankton size-fraction (i.e., <2 µm, mainly cyanobacteria, Supplementary Figure S3) and microplankton (i.e., >20 µm). Picophytoplankton cells were about 20% more abundant in Fe-REG than in the other two treatments by the end of the experiment, and this difference was significant (Student’s test, *p* < 0.01, Figure 4a). We also measured higher cell abundances of nanophytoplankton in Fe-REG treatment starting from day 2 (Figure 4b).

Overall, the pigment diagnostic revealed a positive response of diatom and haptophyte biomass to Fe inputs. As indicated by elevated Fuco and Hex pigment concentrations compared to the initial conditions, diatoms’ biomass increased by 6.2 ± 0.5 (Fe-NO), 5.7 ± 0.2 (Fe-NEW) and 8.2 ± 0.6 (Fe-REG)-fold, while haptophytes’ biomass increased by 1.9 ± 0.1 (Fe-NO), 2.1 ± 0.5 (Fe-NEW) and 2.5 ± 0.3 (Fe-REG)-fold in 6 days. The shift from a haptophyte-dominated community to a mix of haptophytes and diatoms is illustrated by the change in the Fuco/Hex pigment ratio over time (Figure 4c). The fucoxanthin concentration (indicator for diatoms) overtook the 19′-Hex concentration (indicator for haptophytes) after day 4. Interestingly, this shift occurred faster in the Fe-NO and Fe-NEW treatments than in the Fe-REG treatment. However, despite the increase in biomass for these two major phytoplankton groups, their contribution to the overall phytoplankton community decreased. Indeed, while they represented together more than 65% on day 0, diatoms and haptophytes represented 57–59% of the phytoplankton community on day 6 (Figure 4d).

#### 3.2.2. Responses of Heterotrophs

**Figure 5:**
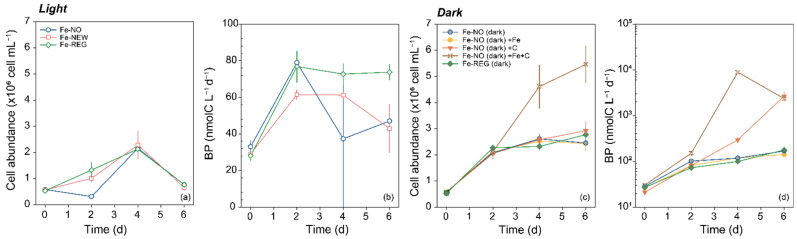
Time course of (**a**) bacterial cell abundance and (**b**) bacterial production (BP) in Fe-NO, Fe-NEW and Fe-REG treatments for incubation under natural daylight cycle and 73 ± 5% of surface irradiance (empty symbols). Time course of (**c**) bacterial cell abundance and (**d**) BP in the dark (filled symbols) for Fe-NO and Fe-REG, and for Fe-NO amended with Fe (brown color, Fe-NO (dark) +Fe),with carbon (orange color, Fe-NO (dark) +C), and both elements (yellow color, Fe-NO (dark) +Fe+C). Note the logarithmic scale for the *y*-axis in (**d**). Error bars represent the standard deviation of triplicate incubation bottles.

For bacteria, we monitored BP and cell abundance in the three treatments and in additional incubations maintained in the dark. We start by describing the results for incubation under daylight cycle (Figure 5). Except for Fe-NO, an increase in cell abundance was noticeable after 2 days of incubation and reached its maximum for all treatments on day 4 before it decreased (Figure 5a). The observed changes were not statistically different from each other, except at day 2, when the cell abundance in the Fe-NO treatment was significantly lower than in the other two (one-way ANOVA, *p* < 0.05). Among bacterial cells, the relative proportion of high (HNA) and low (LNA) nucleic acid content also varied (Supplementary Figure S3). An increase of HNA abundance and a constant number of LNA were noted during the first 4 days of incubation. The proportion of HNA increased from 2–4% at the initial time to 11% (Fe-NO) and 36–43% (Fe-REG and Fe-NEW, respectively) at day 2, followed by an increase of up to 60% by day 4 in all treatments. After day 4, HNA declined and accounted for less than 10% of bacterial cells by the end of the experiment, in all treatments. Similar to what was observed for cell abundance, BP also increased; however, significant differences were found between treatments over the time-course of the experiment. Among all time points and treatments, BP ranged from 28 to 79 (nmolC L^−1^ d ^−1^), but while BP remained constant after day 2 in the Fe-REG treatment, a clear decrease was observed in the Fe-NO and Fe-NEW treatments (Figure 5b). A comparison of cell-specific BP further confirms that heterotrophic bacteria were 1.5 times more active by the end of the experiment in the Fe-REG treatment (9.7 ± 0.9 × 100 fmolC cell^−1^ d^−1^) than in Fe-NO (0.063 ± 0.015 fmolC cell^−1^ d^−1^) and Fe-NEW (0.0676 ± 0.026 fmolC cell^−1^ d^−1^) treatments, and this difference was significant (one-way ANOVA, *p* < 0.05). Interestingly, the decoupling in trends between BP and cell abundance may indicate that LNA cells were mostly active in the Fe-REG treatment. Knowledge of the environmental controls on bacteria is needed to interpret these results in terms of treatments differences. In high-nutrient, low-chlorophyll (HNLC) regions, both DFe and dissolved organic carbon (DOC) may be present at limiting concentrations for heterotrophic bacteria [53,54] which leads not only to interactions between phytoplankton and bacteria [40], but also to interactions between different groups of bacteria to access Fe [55,56]. The primary dependence of bacterial growth on one or the other element also directly influences their interactions with primary producers. To determine whether bacterial growth was constrained by carbon (C), Fe, or both elements under the initial conditions of our experiment, we investigated their responses to Fe, C and concomitant Fe and C additions during parallel incubations (Figure 5). This parallel experiment was conducted for 6 days in the dark; we therefore consider primary production by phytoplankton and the resulting organic enrichment unlikely. Sole additions of Fe or C did not result in any significant enhancement of cell abundance (Figure 5c).

In accordance with these results, BP in the Fe-amended treatment did not differ from the control with no amendment (Fe-NO dark). However, both single (+C) and combined (+Fe+C) additions of DOC significantly stimulated BP (Figure 5d). When normalized to cell abundance, the specific BP rate was maximal when both Fe and C were added and reached 1.71 ± 0.79 fmolC cell^−1^ d^−1^ on day 4 (Supplementary Table S2).

### 3.3. Macronutrients, DFe and Fe-Binding Ligands

The initial macronutrient concentrations were high and therefore not likely to limit the microbial community (Supplementary Table S3). In all treatments, there was little NO_3_ consumption over time, but there was significant NH_4_^+^ drawdown observed by day 2 (Supplementary Figure S4). The drawdown in NH_4_^+^ was accompanied by a notable rise in HNA cells in all three treatments by day 4 (Supplementary Figure S3). Among all incubation bottles and time points, the minimal Si concentration was 2.4 µM, which is well above the limiting levels of <1 µM reached in mid-summer in the Subantarctic [57,58]. In contrast, the final DFe concentrations in all treatments systematically reached limiting levels of ~0.1 nM by the end of the incubations (Table 1), which is consistent with the persistent decline in F_v_/F_m_ (Figure 3).

At the start of the experiment, we measured the highest concentration in total Fe-binding ligands for the Fe-REG treatment (Table 1). These ligands were present in a large excess of total DFe in the Fe-REG treatment (0.26 nM of DFe and 2.04 nM ligands) and were defined predominantly as weak Fe-binding ligands class (L2) with a stability constant log K Fe’L < 12 (Table 1). It should be noted that, contrary to weak ligands, strong Fe-binding ligands (L1; log K Fe’L > 12) may decrease Fe bioavailability and are typically used to define the lower limit of Fe bioavailability in phytoplankton-based uptake assays [59].

## 4. Discussion

The goal of this study was to provide a mechanistic understanding of the biological responses to mixed-layer deepening when biota are acclimated to severe Fe limitation. Over the summer, the *f*e ratio (new Fe/(new Fe + surface recycled Fe) declines and can reach as low as 0.06, meaning that phytoplankton is heavily reliant on DFe recycled in surface waters ([3] and reference herein). Meantime, diapycnal diffusion becomes the predominant physical mechanism of DFe resupply (Figure 1, [3]). In this context we sought to test whether freshly regenerated Fe by particle-attached bacteria can partly support the growth of phytoplankton in surface waters. To discuss the results of this study, we first comment on the biological context of our experiments as we discuss specific phytoplankton responses to changes in DFe concentrations and DFe sources. Then, we discuss the role of bacteria in influencing Fe bioavailability to phytoplankton.

### 4.1. In-Eddy Microbial Residents Survive to Severe Fe-Limitation via Intense Recycling

In the eddy, surface DFe levels were exceedingly low (<50 pM). Remarkedly, while Chl *a* and primary productivity were respectively about 1.5 and 3 times lower than surrounding Subantarctic waters ([36,37]), confirming Fe-limitation by cells within the eddy, the photosynthetic competence was relatively high (F_v_/F_m_ = 0.47 ± 0.07). This suggests that the sampled phytoplankton community was somehow healthy and that cells were highly reliant upon recycled Fe by different members of the microbial community. DFe isotopes confirmed that enhanced bacterially mediated Fe recycling occurred below 100 m depth, and suggested that cells in the euphotic zone also upregulated Fe uptake and recycling [36].

In the Southern Ocean, the pool of biogenic Fe in surface waters can be recycled by the action of grazers [22,23], viruses [60,61] and bacteria [22,40]. Within the mixed layer (0–100 m), zooplankton abundance and biomass were substantially higher within the eddy relative to the edge (Supplementary Figure S1). For bacteria, cell abundance was on average (0–300 m) three times higher at the edge (1.32 ± 0.26 × 10^6^ cells mL^−1^, *n* = 7) than at the center of the eddy (0.43 ± 0.22 × 10^6^ cells mL^−1^, *n* = 5); relative to the total assemblage, the number of HNA bacteria were also found to be higher at the edge (56 ± 11% HNA) while LNA bacteria were more prominent in the center (95 ± 2% LNA). Conversely, BP was the highest at the center of the eddy (Supplementary Figure S5)—more than five times higher than the rates measured at the edge when normalized by cell abundance (Supplementary Figure S5b). This result was unexpected because it challenges the common theory that LNA bacteria are inactive while HNA cells are generally considered the active part of the bacterial group [62]. High specific growth rates for LNA in nutrient-limited waters have contradicted this view in the past [63] and this marked discrepancy between the proportion of HNA and BP rates in our study is additional evidence that LNA bacteria are an active part of microbial communities. One additional explanation is a profound impact of specific grazing by microzooplankton on HNA cells’ abundance [64,65]. Importantly, the BP results demonstrate that intense bacterial activity occurred in the eddy, which may have been supplemented with grazing to result in the rapid recycling of Fe (and the concurrent release of Fe-binding ligands) in the upper surface ocean.

### 4.2. What Phytoplankton Taxa Are Favored by Fe Regenerated from Particles?

The initial phytoplankton community was numerically dominated by the nanophytoplankton fraction (NPF: 65%) among which haptophytes represented a significant portion (81%), while diatoms accounted for 16%. Both haptophytes and diatoms have high Fe requirements and may experience competitive interactions in HNLC regions [66]. The outcome of competition between the two algal groups can potentially influence the carbon export by the biological pump [66,67]. Our study shows that diatoms rapidly outcompete haptophytes in all treatments. These two major groups of phytoplankton seem to adopt different strategies (i.e., r- or K-strategists) for growth. Diatoms (r-strategists) have generally higher growth rates than haptophytes, and consequently may have a competitive advantage in changing environments [67]. This separation in strategies was previously described at the transcriptional level. Diatoms were found to have growth-related transcriptional activity with nutrient enrichments, whereas the activity of haptophytes was decreased [68]. However, haptophyte (K-strategists) also tend to regulate their metal transport systems faster, which may favor them in accessing various forms of Fe. Interestingly, as compared to haptophytes’ biomass, increase in the diatoms’ biomass was the slowest in the Fe-REG treatment, where the initial DFe concentration exceeded the putative DFe threshold of 0.2 nM required for diatoms to alleviate Fe stress [69]. This departure from theory may reflect the inability of large diatoms to outcompete pico- and nanophytoplankton for regenerated Fe. Thus, despite diatoms requiring little Fe to bloom [16,24,70] they could not access enough regenerated Fe to exploit the available macronutrients in the Fe-REG treatment (i.e., 6.07 ± 0.07 µM SiOH_4_, Supplementary Table S3). It is important to note that the pigment fucoxanthin, mainly found in diatoms, can also be found in some haptophyte groups, such as *Phaeocystis.* Several species of *Phaeocystis* increase their fucoxanthin content in response to Fe fertilization (e.g., [45,71]). An unknown part of the increasing fucoxanthin concentration found in our study may therefore result from haptophytes’ instead of diatoms’ growth. Thus, the response of haptophytes may even be more pronounced than described here. The advantage of non-diatom cells (i.e., the picophytoplankton fraction) may be attributed to their physico-chemical properties (e.g., lower surface area: ratio and higher diffusion rates), but it may also be due to the bioavailability of DFe following ligand complexation.

### 4.3. Bioavailability of Fe from Remineralization of Particles

Microbial remineralization of particulate materials supplies ligands which can form complexes with freshly regenerated DFe, keeping it in solution [26,72]. The release of both strong and weak Fe-binding ligands by the heterotrophic community were measured during previous subsurface ocean experiments with marine particles [73,74,75]. The absence of predators during the preparation of regenerated DFe sources (i.e., resuspension in 0.2 µm filtered seawater) would have altered the grazer-mediated release of DFe, likely dominant within the eddy—based on the microzooplankton biomass (Supplementary Figure S1)—and more broadly in the Subantarctic [5,23,28,76,77]. Thus, we cannot rule out that the amount of DFe regenerated may have been less, relative to that in situ, during the incubation of particles with no grazers, but we can reasonably assume that viral abundance was not affected by the resuspension of particles in viral-replete (i.e., <0.2 µm filtered) seawater (e.g., [78]). Therefore, we consider the 16% regeneration rate derived as a lower estimate, especially because a significant amount of the Fe released during the experiment was observed to be rapidly assimilated by the prokaryotes present. This tight coupling between Fe uptake and release also interrogates on the impact of free-living bacteria on the Fe distribution in the ocean interior.

Both large and small cells of phytoplankton can take up new Fe [24], but to what extent large cells can access regenerated Fe was a driven question for this study. Based on the diagnostic pigment, the microphytoplankton fraction increased similarly (+12 ± 0.6% increase by day 6, *n* = 9) in all treatments. This result was not necessarily expected knowing that regenerated DFe may have altered bioavailability during remineralization, for example, due to the degree of complexation to strong Fe-binding L1 class ligands. In our study, there was no detection of type L1 strong ligands by electrochemical analysis. The same analytical technique was employed in [74] but the authors showed that it failed to detect L1 in their samples, although siderophores (which have conditional stability constants comparable to greater than L1 ligands [79] were detected by mass spectrometry. This group of strong ligands likely includes siderophores, which are small molecules produced by bacteria (e.g., [75,80,81]). The ligands associated with particle breakdown also tend to have lower conditional stability constants (log KFe ′L 10–12, or <10, [28,29]). It is probable that particle-associated siderophores were present at very low concentrations in the Fe-REG treatment. However, their contribution to the ligand pool may be too small, when compared to other weaker organic ligands, to be detected by the method we employed. Given that it is not clear what properties of ligands dictate the bioavailability of Fe, it is difficult to draw conclusions here. Yet, it was possible to calculate the uncomplexed Fe (Fe’) concentration, the most bioavailable and scarcest source of DFe [59,82,83], and it is interesting to note that there was 1.7 times more Fe’ in the Fe-NEW treatment, and three times more Fe’ in the Fe-REG treatment than in Fe-NO treatment at the initial time point (Table 1). Although Fe’ concentration represented systematically less than 1% of total DFe, the influence of other members of the microbial community may have contributed to supplement this pool of DFe during the experiment.

### 4.4. Competition between Phytoplankton and Bacteria for Fe: A Misunderstood Story?

**Figure 6:**
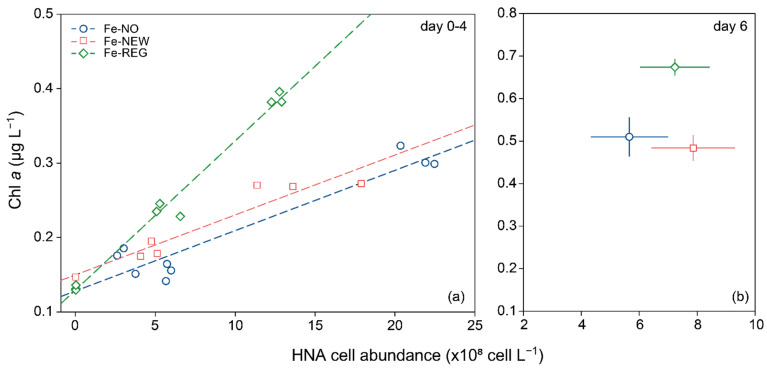
Relationship between Chl *a* concentration and high nucleic content (HNA) bacterial abundance for the three treatments for day 0–4 (**a**) and day 6 (**b**). The best-fit lines of the linear models are plotted (Fe-NO: slope = 8.1 × 10^−11^, R = 0.95, *p* < 0.001; Fe-NEW: slope = 1.2 × 10^−10^, R = 0.95, *p*< 0.001; Fe-REG: slope = 2.0 × 10^−10^, R = 0.99, *p* < 0.001). (**a**) Dots represent the value for each independent replicate (day 0–4). (**b**) Dots and error bars represent the average and standard deviation of triplicate incubation bottles, respectively.

The (sub-saturating) addition of DFe and nutrients with realistic stoichiometries (Table 1 and Table S3), along with the partial relief in grazing pressure following dilution [84], reproduced the perturbations experienced by natural communities during mixed-layer deepening well. Therefore, our results can serve to tease apart the temporal effects of changes in DFe sources on shifts in microbial community composition. From early spring to late summer, the phytoplankton community evolves with the transition from the utilization of new Fe (i.e., winter reserve Fe stocks) to regenerated Fe [24] (Figure 1), which maintains primary productivity. During this transition, fast-growing bacteria can rapidly switch to Fe limitation if phytoplankton-derived organic carbon is available, resulting in an increased need for Fe [39,85] and competition with the pico-nanoplankton size-fraction (2–20 µm) to access the resource [40]. In our study, we observed a strong and positive relationship between HNA cell abundance and increased Chl *a*. This is consistent with previous studies that show increased abundance in HNA cells in response to enhanced phytoplankton-derived organic substrate [86] in areas where bacterial assemblages were predominantly controlled by resources, rather than grazing [87]. However, the preferential response of the phytoplankton biomass relative to HNA cells in the Fe-REG treatment (Figure 6) also suggests that autotrophic cells outcompete with bacteria, and they benefited most from the added DFe.

To explain this result, a sequence of events can be drawn following the evolution of the physiological states of bacteria, which may be reflected by shift from LNA- to HNA-dominated bacterial communities [88]. Since bacteria were initially C-limited, we believe the DFe supply indirectly benefited them through the stimulation of microphytoplankton growth (e.g., diatoms) and the following release of DOC. Then, we hypothesize that HNA bacteria were outcompeted by picophytoplankton for Fe, leading to a return of the LNA-dominance of the bacterial community observed by the end of experiment. When bacteria are Fe-limited, their cell machinery cannot efficiently break down organic molecules. The resulting reduction in energy can impact the cell division ([39]) which is consistent with the marked decreased in the bacterial cells’ abundance (LNA + HNA) found in the incubations. Further, we did not observe this reduction in bacterial cell abundance for the incubation performed in the dark (Figure 5c). Together, these observations support the scenario that picophytoplankton outcompete bacteria for Fe uptake and we propose that this competition is partly explained by the presence of cyanobacteria. Unfortunately, research into interactive co-limitation for C and Fe is still lacking to discuss further this result. Future studies are also needed to clarify the capability of mixotrophy by cyanobacteria as an adaptation to low Fe availability, as well as knowledge on the significance of LNA or HNA cell content in determining the bacterial Fe and carbon demand. Moving forward, future work in modeling may enable researchers to integrate observations of the partitioning of BP specific to HNA and LNA, but also to better interpret interaction between phytoplankton and bacteria.

Theoretical modeling studies generally conclude that bacteria are ruthless competitors for DFe due to their high Fe:C molar ratio requirements, leading to a decrease in phytoplankton biomass in simulations [89,90]. However, observations based on shipboard experiments [31,40], this study point to a more complex scenario for which the outcome is, on the contrary, bacteria being outcompeted by phytoplankton close in size (i.e., 0.2–2 µm, picophytoplankton). The production of siderophores by bacteria in Fe-limited environments is also a recurrent argument to justify an advantage of the latter over phytoplankton. However, the cost of siderophore excretion is metabolically expensive, and especially where organic carbon is limiting [91]. An alternative Fe uptake strategy shared by both microbial groups (bacteria and phytoplankton) better suited our observations—Fe acquisition by means of reduction. Reduction operates on the FeL complex and involves the dissociation of Fe from its chelating ligand followed by transport of Fe’ into the cell. Phytoplankton equipped with this strategy would be able to integrate Fe from a variety of sources, giving them an obvious competitive advantage in Fe acquisition. Cell size, and by extension the number of transmembrane transporters, has a marked influence on accessing Fe’ [59]. This may explain why picophytoplanktons are equally as adept to bacteria at accessing either new or regenerated Fe [28], but does not explain why bacteria seem negatively affected by the presence of picophytoplankton. Part of the answer may lie in the mixotrophic behavior of some members of this size-fraction (e.g., cyanobacteria) as other observations in the SAZ suggest [40].

### 4.5. High Resilience of Microbial Residents Makes the Ferrous Wheel Spins Fast

**Figure 7:**
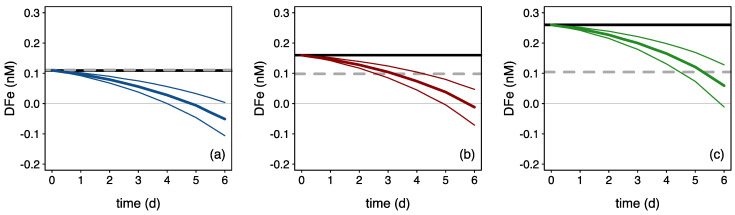
Theoretical evolution of DFe driven by phytoplankton uptake (colored curves) for the (**a**) Fe-NO, (**b**) Fe-NEW, and (**c**) Fe-REG treatments. Theoretical evolutions of DFe are represented by colored lines: blue, red and green for the Fe-NO, Fe-NEW and Fe-REG treatments, respectively, and were generated by combining the in situ size-fractionated Fe uptake rates (Supplementary Table S4) with the evolution of the pico-, nano- and microphytoplankton biomass obtained from a diagnostic pigment criteria (Section 2.2). Mean and standard deviations for the three biological replicates are shown in thick and thin colored lines, respectively. The black and grey-dotted lines are a linear interpolation between the measured initial and final DFe concentrations.

While the algal responses seemed comparable between treatments (Table 1), the ΔDFe/ΔChla ratio (i.e., the drawdown in DFe over the increase in Chl *a*) varied a 10-fold between treatments: 0.03, 0.2 and 0.3 for Fe-NO, Fe-NEW and Fe-REG, respectively. The difference in ΔDFe/ΔChl a between treatments may reflect the high capacity of the in-eddy microbial community to rapidly acquire recycled DFe when external DFe inputs are inadequate to support growth, which we explore further here. To avoid confusion, the term “recycled Fe” refers to the replenishment of the DFe pool due to biotic processes in the incubation bottles, as opposed to the terms “regenerated Fe” or “new Fe” that refer to the source of Fe added to the Fe-REG and Fe-NEW treatments. One way to explore the ability of microbial community members to recycle Fe is to determine the amount of Fe recycled as a function of Fe uptake by phytoplankton. Since the uptake of Fe was not directly measured in our experiment, we computed in situ size-fractionated Fe uptake rates measured on the natural communities during the same study (Supplementary Table S4, [36]) with the development of the pico-, nano- and microphytoplankton biomass obtained from a diagnostic pigment criterion (Figure 7). The difference between the final (measured) and theoretical concentrations of DFe in the incubation bottles thus represents the cumulative amount of DFe used to support phytoplankton growth (Figure 6). By day 6, this quantity equals 163, 110 and 45 pmol Fe, which represents 148, 69 and 17% of the DFe initial concentration in the Fe-NO, Fe-NEW and Fe-REG treatments, respectively. Although variations in Fe uptake rates or the use of Fe stored intracellularly might have impacted the accumulation or release of DFe (e.g., [92,93], this exercise highlights a potentially wide range in the proportion of recycled Fe used by phytoplankton to grow: 59% (Fe-NO), 41% (Fe-NEW) and 15% (Fe-REG). This result provides support for the intense microbially mediated Fe recycling inferred from Fe stable isotope signatures [36]. In the control (Fe-NO), DFe concentration differed little between the initial and final time-points, suggesting a tight coupling between Fe uptake and Fe recycling rates. Perhaps surprisingly, this rapid in situ turnover time of the biotic Fe pool occurred in the absence of grazing pressure in our experimental set-up (dilution effect). Equally striking within our datasets is the similar level of DFe (0.10 ± 0.01 *n* = 9) measured in all the bottles by the end of the experiment despite a 1.4 to 2.4-fold difference at the initial time (Supplementary Table S3). After 6 days of incubation, we believe this result—from nine independent incubation bottles—cannot be random or a coincidence. Instead, we assume that the microbial assembly is very efficient in returning to a “steady state”. When scaled to the in-eddy inventory, such a high resilience in the ferrous wheel is inextricably linked to the timescale and efficiency of Fe recycling. Thus, the establishment of a linkage between Fe chemistry and biological interactions is critical to predict the extent of phytoplankton blooms in a changing ocean.

## 5. Conclusions

Two main conclusions can be drawn from this study. First, independent lines of evidence are presented which validate intense recycling by an acclimated surface community. The present study further confirms that bacterially mediated Fe plays a crucial role in planktonic growth. Both the addition of Fe-NEW and Fe-REG stimulated the community, but our results support the idea that freshly regenerated materials (Fe-REG) may be a better source of bioavailable Fe than more aged or more crystalline forms (as compared to Fe-NEW). The challenging question that emerges is what chemical modifications are encountered by this regenerated Fe during transport from the meso- to epipelagic zone, and what processes (and at what rate) control Fe bioavailability during that transport. We showed that DFe regenerated from particles and new DFe were not identically beneficial to all phytoplankton taxa at same rate. Therefore, these transformations can drive shifts in phytoplankton community composition that can alter how much organic carbon can be exported to the deep ocean. Second, we also show that resident cells—with low *f*e ratios at the end of the summer—can rapidly acclimate to different Fe sources. This ability may be due in part to the competitive advantage of small cells (prokaryotes and picophytoplankton) to access DFe. These interspecific interactions, exacerbated by the partial relief in grazing pressure driven by the dilution from ML deepening, may prevent diatoms from outgrowing other phytoplankton taxa and favor the rapid remobilization of intracellular Fe within the ferrous wheel. In addition, the plasticity of the autotrophic metabolic machinery and the potential role played by bacteria, as a major component of the biotic Fe pool, can further limit the effect of vertical Fe supply. Together, these mechanisms buffer the response of phytoplankton biomass to vertical Fe supply despite extremely low ambient DFe levels.

## Figures and Tables

**Table 1:** Initial and final concentrations in dissolved iron (DFe), inorganic Fe (Fe’), total iron-binding ligand (L_T_), and conditional stability constants (log K’_Fe’L_). Values within parentheses are the standard deviation of the mean of three measurements. ‘ND’ denotes no data.

	DFe (nM)		L_T_ (nM)		Log K’_Fe’L_		Fe’ (pM) *	
Treatment	Initial	Final	Initial	Final	Initial	Final	Initial	Final
Fe-NO	0.11 (0.01)	0.11 (0.01)	1.36 (0.13)	ND	11.0 (0.3)	ND	0.80 (0.1)	ND
Fe-NEW	0.16 (0.04)	0.09 (0.01)	1.69 (0.21)	ND	10.8 (0.3)	ND	1.49 (0.4)	ND
Fe-REG	0.26 (0.02)	0.10 (0.01)	2.04 (0.11)	ND	10.7 (0.2)	ND	2.52 (0.1)	ND

* L_T_ and log K’_Fe’L_ were determined using cathodic stripping voltammetry and Fe’ was calculated by Fe′ = DFe/[(L_T_ − DFe) × K’_Fe’L_].

## Data Availability

Size-fractionated Fe uptake rates are from [36].

## Supplementary Materials

The following supporting information can be downloaded at: https://www.mdpi.com/article/10.3390/microorganisms10081655/s1, Figure S1: (a) Temperature, (b) chlorophyll a (Chl a) concentration, and zooplankton (c) abundance and (d) biomass (obtained from a Laser Optical Plankton Recorder) within the cold-core eddy and at the eddy’s periphery; Figure S2: A schematic representation of the experimental set-up; Figure S3: Time course of the (left) relative abundance of high nucleic acid (HNA) cells (i.e. HNA/(total bacterial cells) and (right) abundance cyanobacteria measured by flow cytometry; Figure S4: Time course of dissolved inorganic (a) nitrate, (b) nitrite, (c) ammonium, (d) silicate, and (e) phosphate concentrations during the incubation; Figure S5: Depth profiles of bacterial production and abundance at the center and at the edge of the eddy. Profiles of volumetric (a) and cell-specific (cell abundance normalised) bacterial production (b) versus depth; Table S1: Assignments of the seven pigments used in the diagnostic pigments (DP) to phytoplankton group and size-fractions; Table S2: Range of specific bacterial production rates in incubations performed in the dark for Fe-NO and Fe-REG treatments, and Fe-NO treatment amended with DFe (+Fe), DOC (+C) or both (+Fe+C); Table S3: Initial biogeochemical conditions for the Fe-NO, Fe-NEW, and Fe-REG treatments; Table S4: Iron and carbon uptake rates for the different size fractions of the in-eddy phytoplankton community (from [36]).
