# Supplementary material for: Phytoplankton Responses to Bacterially Regenerated Iron in a Southern Ocean Eddy
Source: Microorganisms. 2022 Aug 16;10(8):1655. doi: 10.3390/microorganisms10081655 (PMC9413495; doi:10.3390/microorganisms10081655)
Supplement: Supplementary file 1 [file microorganisms-10-01655-s001.zip › microorganisms-1828973-supplementary.pdf]

microorganisms
MDPI

Supplementary Materials

Supplementary Table S1. Assigments of the seven pigments used in the diagnostic pigments (DP) to phytoplankton group and size-fractions.

**Diagnostic pigment**	**Abbreviation**	**Phytoplankton group(s)**	**Pico (PPF)**	**Nano (NPF)**	**Micro (MPF)**
Fucoxanthin	Fuco	Diatoms, Dinoflagellates			x
Peridinin	Peri	Dinoflagellates			x
19'-Hexanoyloxy-fucoxanthin	Hex	Haptophytes		x	
19'-Butanoyloxy-fucoxanthin	But	Prymnesiophytes, Silicoflagellates		x	
Alloxanthin	Allo	Crytophytes		x	
Total chlorophyll b	TChlb	Euglenoids, Chlorophytes	x		
Zeaxanthin	Zea	Cyanobacteria	x		
**Total diagnostic pigments**	**DP**	All groups considered			

Supplementary Table S2. Range of specific bacterial production rates in incubations performed in the dark for Fe-NO and Fe-REG treatments, and Fe-NO treatment amended with DFe (+Fe), DOC (+C) or both (+Fe+C). Values represented average of three independent replicates and the time point (d) associated.

		**Specific BP (x100 fmolC cell^{-1} d^{-1})**		
Treatment	Nutrients addition	min	max	end of incubation
Fe-NO (dark)	none	4.55 (d0)	5.77 (d6)	5.77
Fe-NO (dark)	+Fe	4.50 (d2)	6.85 (d6)	6.85
Fe-NO (dark)	+C	3.65 (d0)	91.6 (d6)	91.6
Fe-NO (dark)	+Fe+C	5.22 (d0)	192 (d5)	43.2
Fe-REG (dark)	none	3.19 (d2)	6.40 (d6)	6.40

Supplementary Table S3. Initial biogeochemical conditions for the Fe-NO, Fe-NEW, and Fe-REG treatments.

	**Fe-NO**	**Fe-NEW**	**Fe-REG**
Ammonium (µM)	1.22 ± 0.16	0.72 ± 0.24	1.03 ± 0.34
Nitrate (µM)	22.36 ± 0.76	23.26 ± 0.17	23.20 ± 0.03
Nitrite (µM)	0.35 ± 0.07	0.44 ± 0.01	0.45 ± 0.01
Phosphate (µM)	1.62 ± 0.06	1.68 ± 0.01	1.69 ± 0.01
Silicate (µM)	3.16 ± 0.08	5.43 ± 0.01	6.07 ± 0.07
Dissolved iron (nM)	0.11 ± 0.01	0.16 ± 0.04	0.26 ± 0.02
Chlorophyll-a (µg L^{-1})	0.154 ± 0.016	0.132 ± 0.015	0.132 ± 0.003
F_v/F_m	0.52 ± 0.05	0.50 ± 0.05	0.44 ± 0.04

Supplementary Table S4. Iron and carbon uptake rates for the different size fractions of the in-eddy phytoplankton community (from Ellwood et al. 2020). Values within parentheses represent 1 s.d. for replicate measurements.

	0.2-2-µm [a,b]	2-20-µm [a,b]	>20-µm [a,b]
Fe uptake (pmol L^{-1} d^{-1})	17.4 (5.6)	4.6 (3.0)	4.9 (1.2)
C uptake (µmol L^{-1} d^{-1})	0.06 (0.01)	0.05 (0.03)	0.04 (0.01)
Fe:C ratio (µmol mol^{-1})	285.4 (92.5)	99.6 (88.9)	120.6 (43.3)

[a] incubation at 80% incident irradiance; [b] extracellular Fe removed using Ti(III) EDTA-citrate

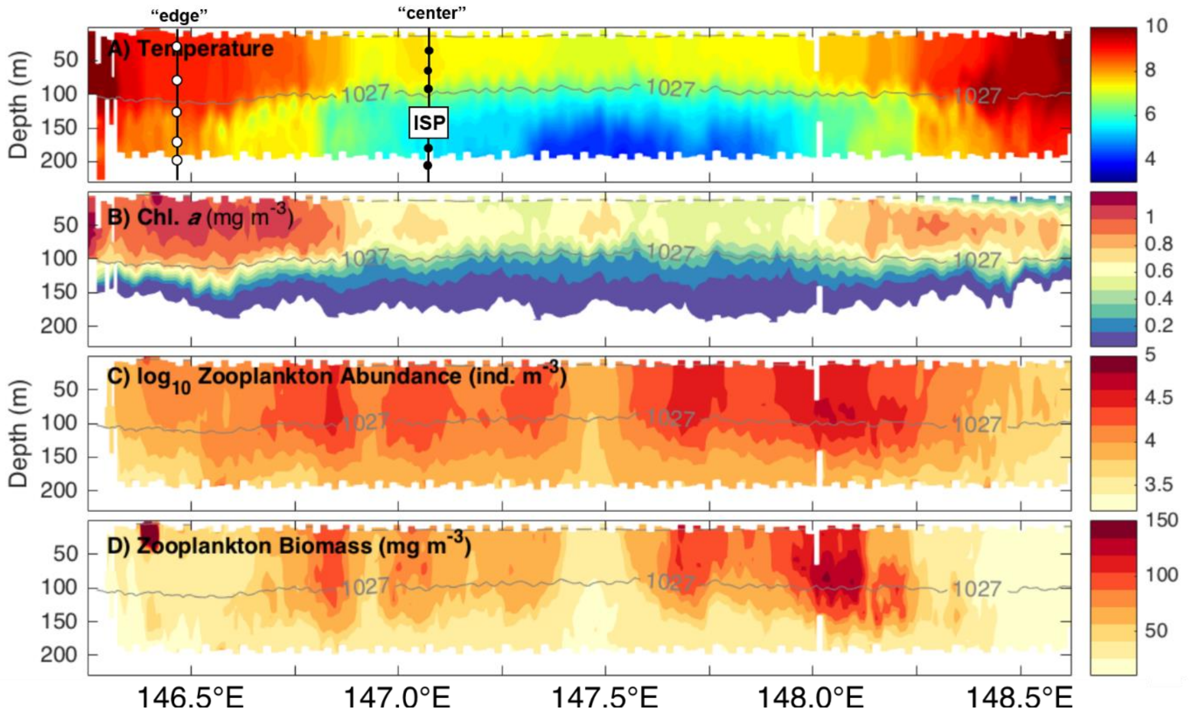

Supplementary Figure S1 (**a**) Temperature, (**b**) Chl *a* concentration, and zooplankton (**c**) abundance and (**d**) biomass (obtained from a Laser Optical Plankton Recorder) within the cold-core eddy and at the eddy's periphery. Location of the sampling for bacterial production profiles at the periphery ("edge", white dots) and at the within the eddy ("center", black dots) are shown in panel (a). Sampling for the surface (5m) microbial community was done at the same location within the eddy ("center") as the collection of subsurface particles by In Situ Pump (ISP) deployed at 150m depth.

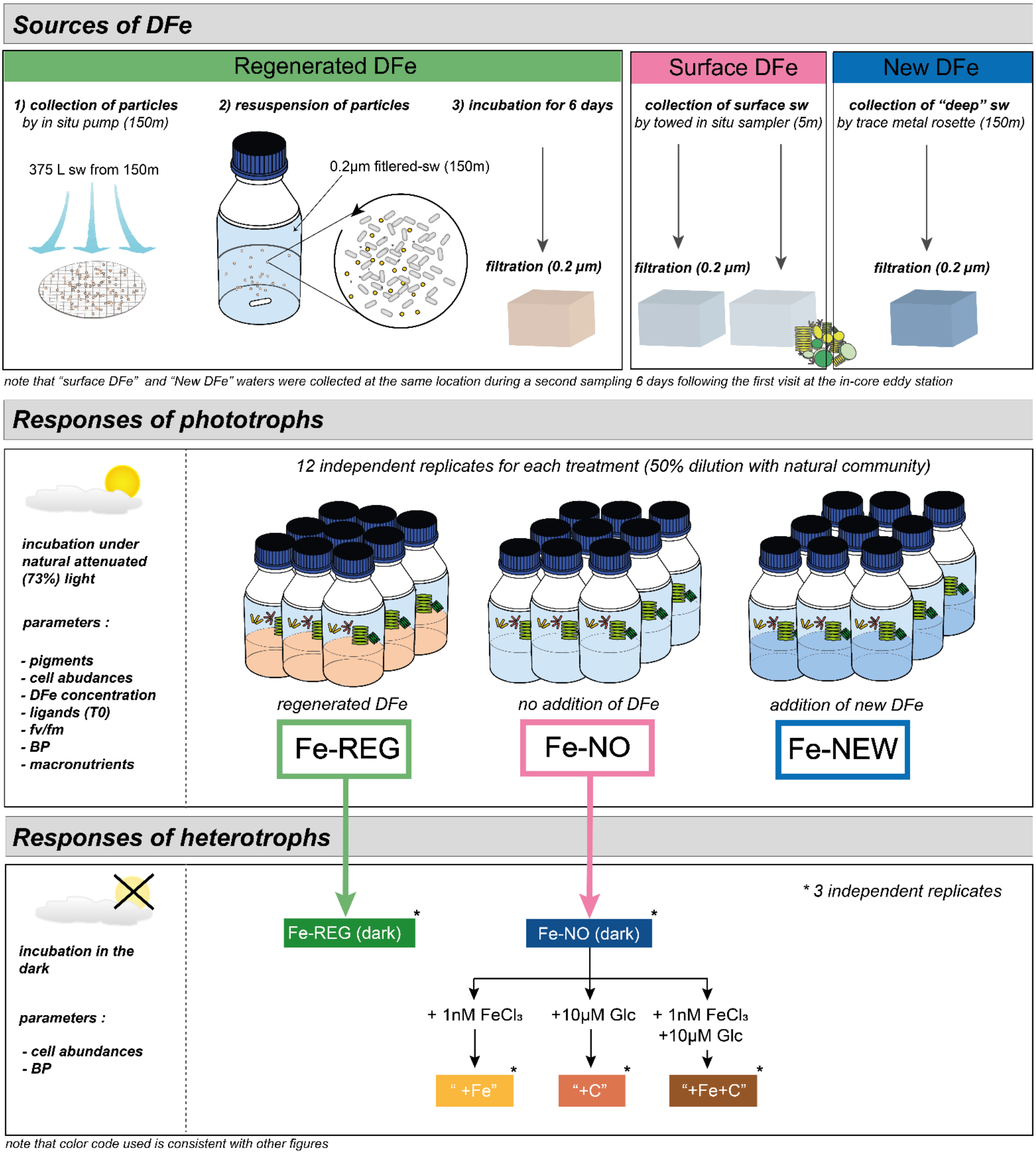

Supplementary Figure S2. A schematic representation of the experimental set-up.

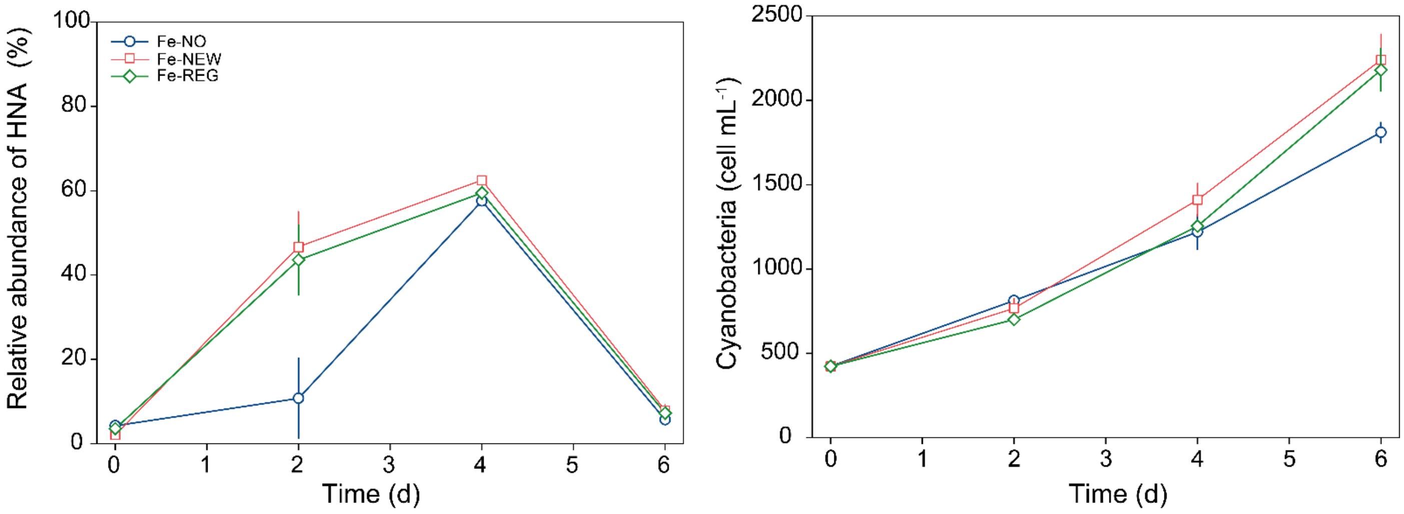

Supplementary Figure S3. Time course of the (left) relative abundance of high nucleic acid (HNA) cells (i.e. HNA/(total bacterial cells) and (right) abundance cyanobacteria measured by flow cytometry. Error bars represent the standard deviation of triplicate incubation bottles.

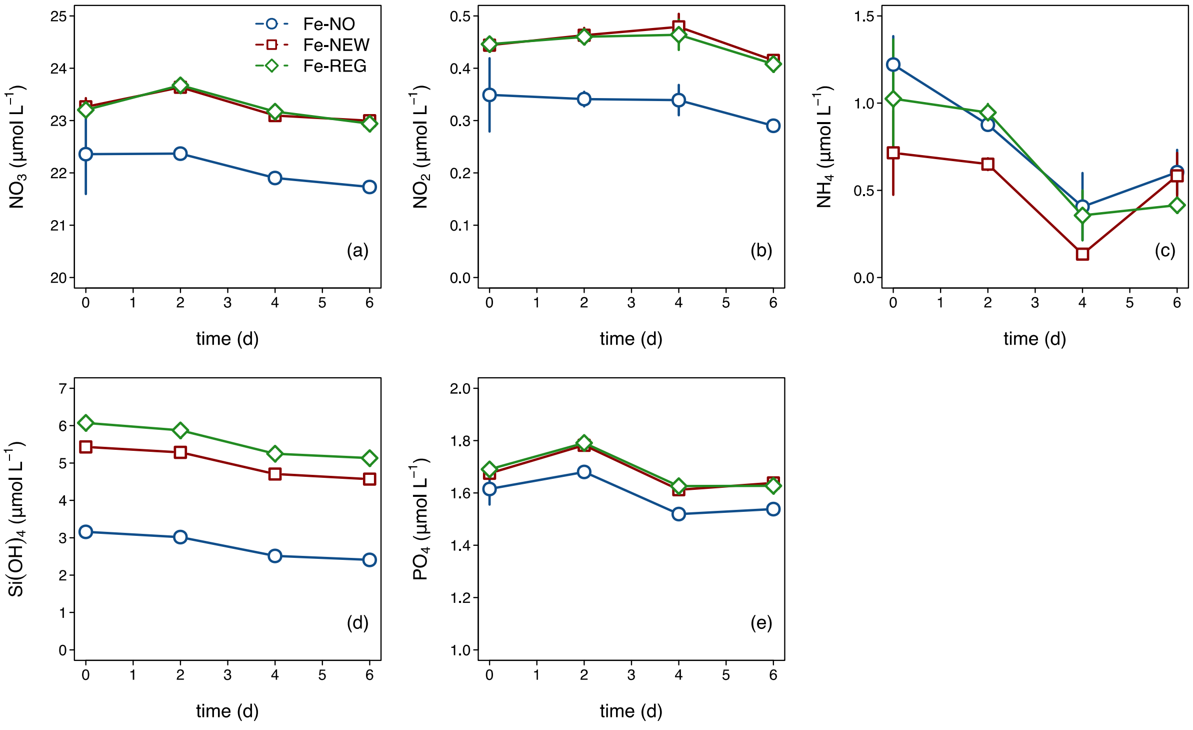

Supplementary Figure S4. Time course of dissolved inorganic (**a**) nitrate, (**b**) nitrite, (**c**) ammonium, (**d**) silicate, and (**e**) phosphate concentrations during the incubation. Error bars represent the standard deviation of triplicate incubation bottles.

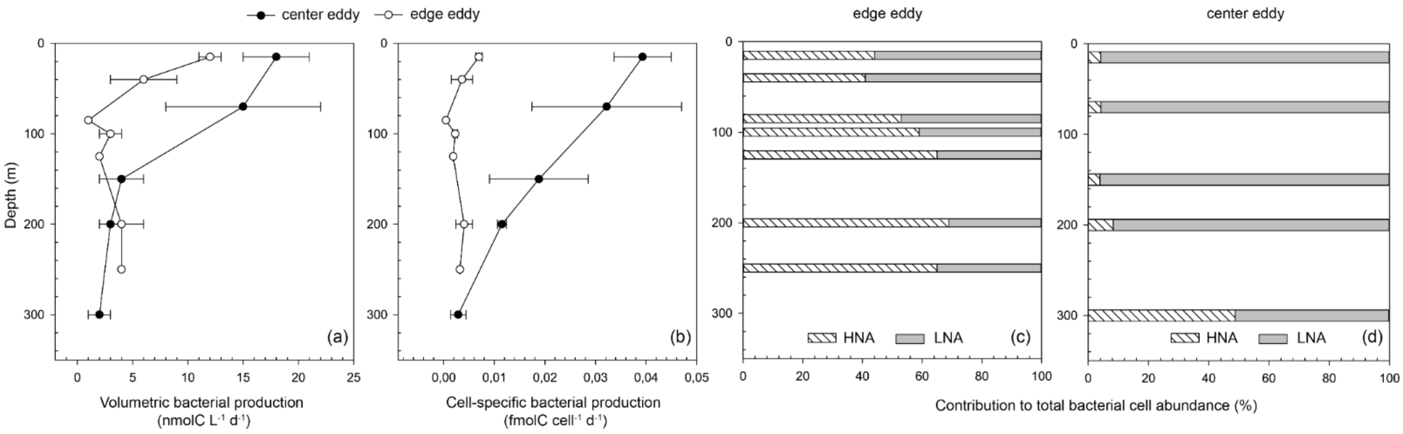

Supplementary Figure S5. Depth profiles of bacterial production and abundance at the center and at the edge of the eddy. Profiles of volumetric **(a)** and cell-specific (cell abundance normalised) bacterial production **(b)** versus depth. Error bars represent 1 standard deviation for replicate measurements. Relative contribution (%) of high DNA content (HNA) and low DNA content (LNA) cells at the edge **(c)** and center **(d)** of the eddy.
